# An Oligodeoxynucleotide That Induces Differentiation of Bone Marrow Mesenchymal Stem Cells to Osteoblasts *in Vitro* and Reduces Alveolar Bone Loss in Rats with Periodontitis

**DOI:** 10.3390/ijms13032877

**Published:** 2012-03-05

**Authors:** Yuqin Shen, Zhiyuan Feng, Chongtao Lin, Xu Hou, Xueju Wang, Jing Wang, Yongli Yu, Liying Wang, Xinhua Sun

**Affiliations:** 1 Department of Periodontics, School of Stomatology, Jilin University, 1500 Qinghua Road, Changchun 130021, China; E-Mails: shen_yuqin@163.com (Y.S.); linchongtao@163.com (C.L.); 2 Department of Orthodontics, People’s Hospital of Shanxi, 29 Shuangta Road, Taiyuan 030012, China; E-Mail: fzy00@sina.com; 3 Department of Orthodontics, School of Stomatology, Jilin University, 1500 Qinghua Road, Changchun 130021, China; E-Mails: houxu_112@126.com (X.H.); 33817805@qq.com (J.W.); 4 Department of Pathology, China-Japan Union Hospital, Jilin University, 1500 Qinghua Road, Changchun 130021, China; E-Mail: wxj20032006@yahoo.com.cn; 5 Department of Immunology, Medical College of Norman Bethune, Jilin University, 1500 Qinghua Road, Changchun 130021, China; E-Mail: ylyu@mail.jlu.edu.cn; 6 Department of Molecular Biology, Medical College of Norman Bethune, Jilin University, 1500 Qinghua Road, Changchun 130021, China

**Keywords:** oligodeoxynucleotide, bone marrow mesenchymal stem cells, osteoblasts, differentiation, periodontitis

## Abstract

To investigate the effect of oligodeoxynucleotides (ODNs) on the differentiation of rat bone marrow mesenchymal stem cells (BMSCs) to osteoblasts, in order to find a candidate ODN with potential for the treatment of periodontitis, a series of ODNs were designed and selected to test their effect on the promotion of the differentiation of BMSCs to osteoblasts *in vitro* and on the repair of periodontal tissue in rats with periodontitis. It was found that MT01, one of the ODNs with the sequences of human mitochondrial DNA, stimulated the proliferation of BMSCs, the differentiation of BMSCs to osteoblasts and mRNA expression of bone-associated factors including Runx2, Osterix, OPG, RANKL and collagen I *in vitro. In vivo* study showed that MT01 prevented the loss of alveolar bone in the rats with periodontitis and induced the production of proteins of OPG and Osterix in the bone tissue. These results indicated that MT01 could induce differentiation of BMSCs to osteoblasts and inhibit the alveolar bone absorption in rats with periodontitis.

## 1. Introduction

Periodontitis is an inflammatory disease which manifests clinically as loss of the supporting periodontal tissues including the periodontal ligament, cementum and alveolar bone. Periodontal therapy is aimed at achieving the complete regeneration of these structures, especially the alveolar bone [1]. To date, several methods have been employed to achieve this goal, e.g., using various bone grafts, growth factors and barrier membranes [2,3]. Generally, the regeneration of alveolar bone relies on the differentiation of bone marrow mesenchymal stem cells (BMSCs) or periodontal ligament stem cells into osteoblasts, which are responsible for the formation of new bone [4,5]. Obviously, inducing and activating more osteoblasts can promote the regeneration of destroyed alveolar bone. To realize this aim, great efforts have been made to induce osteoblasts *in vitro* [1,6]. As the periodontal ligament cells are hard to culture, BMSCs, which can be easily isolated and cultured, are usually selected as the seed cells for generating osteoblasts *in vitro* [7]. To develop agents for preventing the loss of alveolar bone, rats like Wistar rat or Sprague-Dawley rat are often used as animal models because the periodontal anatomy in the molar region of the rats shares similarities with that of humans. By placing ligature in the gingival sulcus around the molar teeth, experimental periodontitis with alveolar bone loss could be readily induced in the rats [8–10].

During the life of periodontium, the alveolar bone continuously remodels its shape in response to both the mechanical forces on the tooth and inflammation [11]. Growth and the modeling/remodeling of the alveolar bone are integral processes including multiple feedback loops between osteoblast and osteoclast [6,12]. The recruitment of new osteoclasts is dependent on the balance between the receptor activator of the NF-κB ligand (RANKL) and osteoprotegerin (OPG) in the osteoblasts [13,14]. The balance determines the formation and activity of osteoclasts. The activated osteoclasts comprise an integral component of bone destruction [15–17]. In addition to OPG and RANKL, runt-related transcription factor 2 (Runx2), Osterix and type I procollagen (collagen I) are also involved in bone formation [18–20]. Currently, the application of various regenerative biomaterials, such as bone autografts, allografts, cell occlusive barrier membranes used in guided tissue regeneration procedures, applications of bone morphogenetic protein (BMP) and growth factors (e.g., enamel matrix proteins), or their combinations, have been pursued with varying degrees of success to regenerate the lost tooth support [21,22]. However, these therapeutic strategies have been shown to be limited in the predictability of healing and in regenerative response in modern clinical practice.

In the recent decade, synthesized single stranded oligodeoxynucleotide (ODN) has been demonstrated to modulate osteoblasts and osteoclasts. CpG containing oligodeoxynucleotides (CpG-ODNs) inhibit the activity of the physiological osteoclast differentiation factor RANKL in early osteoclast precursors (OCPs) but strongly stimulate osteoclastogenesis in cells primed by RANKL. The enhanced osteoclastogenic effect is mediated by TNF-α mediates by an autocrine mechanism [23,24]. The inhibitory effect could suggest the possibility of using CpG-ODNs to block pathological bone loss as in periodontitis [25]. The osteoclastogenic effect of CpG-ODN is dependent on activation of Toll-like receptor 9 (TLR9) as shown in TLR9-deficient (TLR9−/−) mice. Activation of TLR9 in bone marrow-derived osteoclast precursors is more crucial to induction of osteoclastogenesis than activation of the osteoblastic TLR9 [26]. The CpG ODN induced TLR9 signals are transmitted through ERK, p38 and NFκB pathways which are inhibited by chloroquine, suggesting a requirement for endosomal maturation/acidification, the classic CpG ODN mode of action [27]. In addition to TNF-α, IL-12 induced by CpG-ODN mediated TLR9 activation opposes RANKL-induced osteoclast differentiation [28]. In our preliminary studies, we found that MT01 [29], a synthetic single stranded ODN, whose design is based on human mitochondrial DNA, had a significant impact in facilitating osteogenic proliferation and activation. This provided direct evidence for the notion that single strand ODN could regulate the balance of bone formation and resorption, and thus was of great potential in the rebuilding of alveolar bone [30]. However, the effects of ODNs including MT01 on the proliferation and differentiation of BMSCs to osteoblasts have not been clearly elucidated.

In this study, a batch of ODNs, whose design is based on the sequences in human microsatellite DNA and mitochondrion DNA and confirmed with immuno-stimulatory or immuno-inhibitory activities, were screened for their capacity to induce proliferation and differentiation of rat BMSCs. In the process, an ODN, designated as MT01, was found to strongly activate the differentiation of rat BMSCs *in vitro* and significantly reduce the alveolar bone loss in rats with periodontitis.

## 2. Results and Discussion

### 2.1. Screening of ODNs Capable of Stimulating the Proliferation and Differentiation of Rat BMSCs

**Figure 1:**
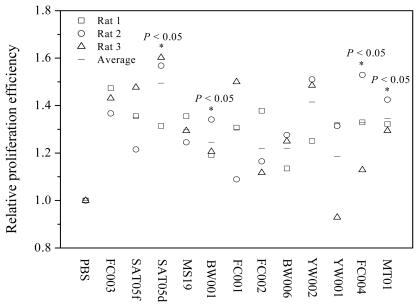
Effect of oligodeoxynucleotides (ODNs) with different sequences on the proliferation of rat bone marrow mesenchymal stem cells (BMSCs). The third passage BMSCs from three different rats were separately seeded in a 96-well-plate at 5 × 10^3^/well and cultured in DMEM for 12 h. After adding ODNs (1 μg/mL, final concentration), the cells were cultured for 72 h and then subjected to MTT assay. OD570 value was used to express the cell proliferation (*n* = 4 per group). * Indicates statistically significant difference (*P* < 0.05) between experimental and control groups.

To detect the effects of ODNs on the proliferation of BMSCs, we selected and synthesized 12 ODNs with different sequences using DNA synthesizer as described in the Experimental Section and tested them for their ability to stimulate the proliferation of rat BMSCs *in vitro*. These ODNs were designed based on the sequences in human microsatellite DNA and mitochondrion DNA and confirmed with immuno-stimulatory or immuno-inhibitory activities [29,31–34]. In the test, Wistar rat BMSCs at the third passage were cultured with a medium containing various ODNs at a final concentration of 1 μg/mL for 72 h. The proliferation of BMSCs was determined using MTT assay. As shown in Figure 1, four ODNs, designated as MT01, SAT05d, BW001 and FC004 were found to have the ability to stimulate the proliferation of all the rat BMSCs. The efficacy was significantly stronger than that induced by PBS (*P* < 0.05, *n* = 4).

**Figure 2:**
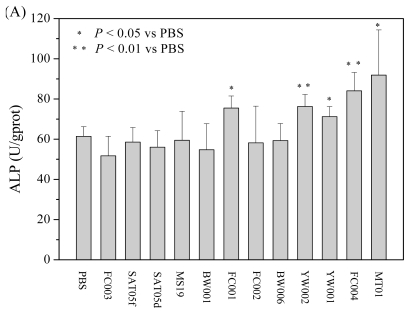

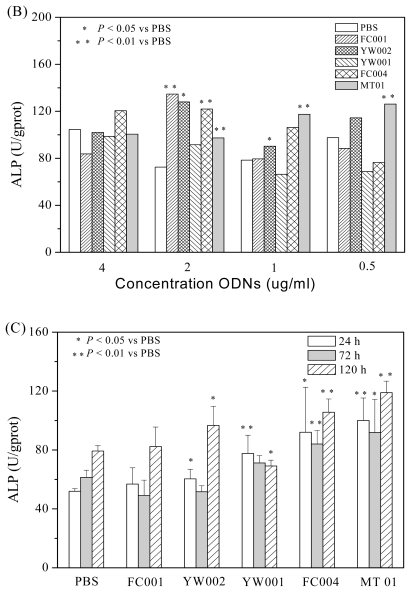
Effect of ODNs with different sequences on the expression of alkaline phosphatase (ALP) in rat BMSCs. (**A**) Screen of the ODNs (*n* = 6 per group); (**B**) Dose effect of the ODNs (*n* = 6 per group); (**C**) Kinetic effect of the ODNs. * Indicates statistically significant difference (*P* < 0.05) between MT01 and PBS; ** Indicates statistically significant difference (*P* < 0.01) between MT01 and PBS.

Accumulating studies showed that BMSCs could be induced to differentiate into osteogenic cells. To observe whether the ODNs could also impact the osteoblast differentiation from BMSC, rat BMSCs, cultured to passage 3, were seeded to 12 well plate (2 × 10^4^ cells per well) in 1000 μL complete DMEM with osteogenic media and then cultured for 24 h. After adding the 12 ODNs (1 μg/mL, final concentration) separately, the cells were cultured for another 72 h and then tested for their expression of alkaline phosphatase (ALP), a marker for the early stage differentiation of osteoblasts. As shown in Figure 2A, MT01, FC001, YW002, YW001 and FC004 could strongly stimulate the differentiation of rat BMSCs to osteoblasts. Based on this, the five ODNs were selected to test their dose-effect on the differentiation dynamically. As described above, the BMSCs at passage 3 were cultured with MT01, FC001, YW002, YW001 or FC004 at a dose range of 0.5, 1, 2 and 4 μg/mL(final concentration), respectively, and then assayed for their ALP activity. It was found that MT01 at 2 μg/mL, 1 μg/mL and 0.5 μg/mL, but not at 4 μg/mL, could induce significant expression of ALP (Figure 2B). Next, the five ODNs were tested for their promotion on the differentiation kinetically. The BMSCs at passage 3 were cultured with each of the ODNs at 1 μg/mL (final concentration) for 24, 72 or 120 h, respectively, and then assayed. The results showed that MT01 and FC004 could significantly induce ALP expression at three time-points (Figure 2C). Overall, the dose and kinetic analysis showed that MT01 was the best candidate ODN to induce the differentiation from BMSCs to osteoblasts.

### 2.2. MT01 Induces the Differentiation of BMSCs to Osteoblasts and Its Possible Mechanism

From the above results, we selected MT01 to study the possible mechanism underlying its effect on inducing the differentiation of BMSCs to osteoblast. As reported, MT01 (5′-ACC CCC TCT ACC CCC TCT ACC CCC TCT-3′), a 27-mer ODN, was synthesized with reference to human mitochondrial DNA. Functionally, MT01 is able to inhibit the proliferation of human peripheral blood mononuclear cells (PBMCs) induced by CpG-ODNs and the production of type I interferon (IFN) from human PBMCs stimulated by TLR agonists, including inactivated influenza virus, imiquimod, inactivated herpes simplex virus-1 (HSV-1) and CpG ODNs. Presumably, the MT01 could be a biological candidate with therapeutic use in TLR activation associated diseases [29]. To investigate the role of MT01 on the differentiation of BMSCs to osteoblast and its possible mechanism, using quantitative real-time PCR, we detected the effects of MT01 on inducing mRNA expression of bone-related factors including RANKL, OPG, Runx2, Osterix and collagen I in BMSCs. The expression of these genes was confirmed to be related to osteoblastogenesis and was required for the osteoblast differentiation [35–37].

**Figure 3:**
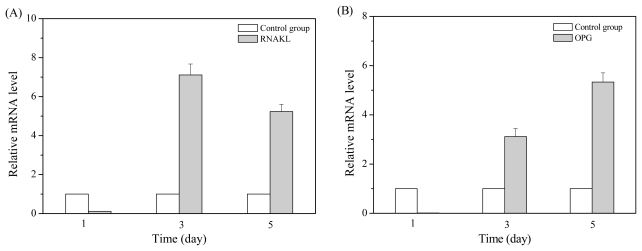

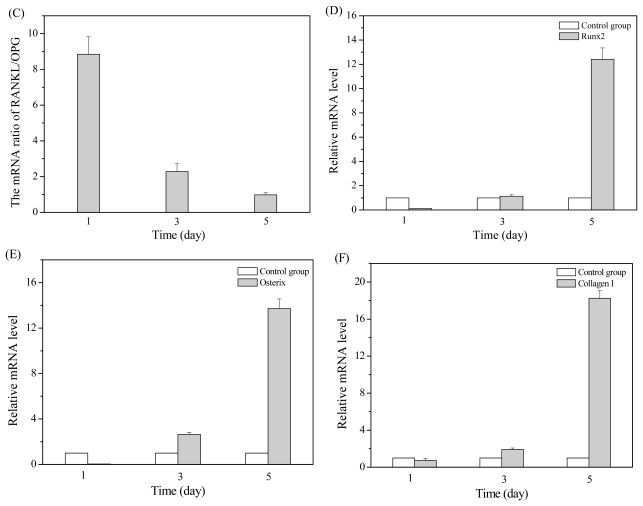
MT01 induced mRNA expression in BMSCs. Rat BMSCs were stimulated with MT01 (1 μg/mL) or PBS for 1, 3 or 5 days. The mRNA expression of the genes related to osteoblastogenesis was detected using quantitative real-time PCR. (**A**) RANKL mRNA expression; (**B**) OPG mRNA expression; (**C**) Ratio of RANKL to OPG mRNA expression; (**D**) Runx2 mRNA expression; (**E**) Osterix mRNA expression; (**F**) Collagen I mRNA expression. β-Actin was used as the internal reference. Data from one representative experiment of three are shown.

In the detection, BMSCs at the third passage were cultured with MT01 at 1 μg/mL (final concentration) for 1, 3 or 5 days, respectively, and their mRNAs were isolated for real-time PCR analysis. The results showed that stimulation with MT01 for 3 or 5 days induced BMSCs to express significantly increased mRNA level of RANKL and OPG (*P* < 0.01) (Figure 3A,B). Noticeably, the mRNA ratio of RANKL/OPG gradually decreased from 1 to 5 days (Figure 3C). Meanwhile, mRNA levels of Runx2, Osterix and collagen I were increasingly induced by MT01 for 3 and 5 days (*P* < 0.01 or *P* < 0.05) (Figure 3D–F). In conclusion, ODN MT01 could significantly promote the differentiation of rat BMSCs to osteoblastic cells and enhance the osteoblast activity, possibly by inducing the up-regulation of Runx2, Osterix, OPG, RANKL and collagen I in the BMSCs.

### 2.3. MT01 Mediated Reduction of Alveolar Bone Loss in Experimental Periodontitis Rats

The purpose of this study was to find the active substance capable of promoting the osteoblastic differentiation of BMSCs, with a potential to be used in tissue repairing and alveolar bone regeneration. To further test whether MT01 could promote the alveolar bone regeneration *in vivo*, we firstly established a rat model of experimental periodontitis using the silk ligation method [8,9]. Immediately after the ligation, the rats were injected once with MT01 (20 μg/kg) into the local periodontal tissue. On days 3, 5, and 7 after the ligation, the rats received three more injections of MT01 (20 μg/kg), respectively. Two or four weeks later, after the ligation, the rats were sacrificed for detecting immunohistochemical changes and alveolar bone loss.

**Figure 4:**
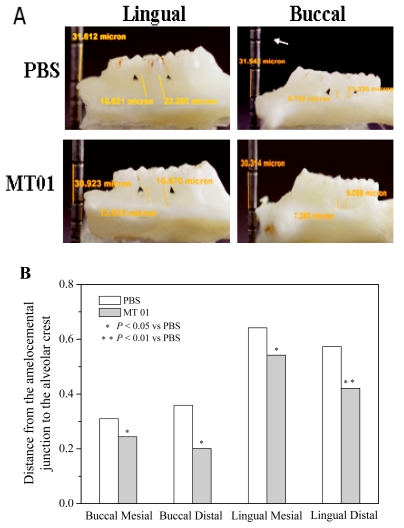
Influence of MT01 on alveolar bone loss in rat with periodontitis. Macroscopic aspects (Lingual and Buccal view) of the mandibles were observed. The yellow lines represented the distance from the amelocemental junction to the alveolar crest. (**A**) Analysis of the alveolar bone loss by SPOT RT software v3.5 [38,39]; (**B**) The extent of alveolar bone loss showed in the change of measurement between the control and MT01 treatment groups where *n* = 3 per group. * Indicates statistically significant difference (*P* < 0.05) between MT01 and PBS. ** Indicates statistically significant difference (*P* < 0.01) between MT01 and PBS.

The alveolar bone loss was determined by measuring the distance from the amelocemental junction to the alveolar crest. The results showed that the absorption of alveolar bone had no significant difference between the MT01 treated rats and PBS treated rats at 2 weeks. However, at 4 weeks after the ligation, the absorption of alveolar bone, especially of palatal bone, in the MT01 treated rats was dramatically reduced compared to that in the PBS treated rats (*P* < 0.05) (Figure 4A,B). Through analysis of the extent of alveolar bone loss, it could be concluded that MT01 could prevent alveolar bone loss.

**Figure 5:**
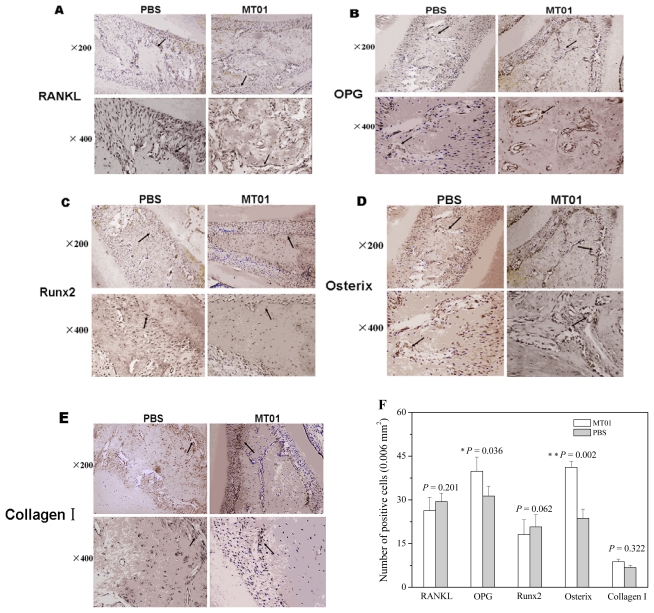
Effect of MT01 on the production of proteins of bone-associated factors. In the sections of the alveolar bone, RANKL (**A**), OPG (**B**), Runx2 (**C**), Osterix (**D**) and collagen I (**E**) producing cells were recognized by their specific antibodies, respectively. The positive cells were analyzed statistically and shown in **F**. Data were expressed by mean ± SD of 6 rats each group (* *P* < 0.05; ** *P* < 0.01, compared with the PBS group).

Immunohistochemical detection (Figure 5) showed that MT01 treatment did not elevate the production of RANKL, Runx2 and collagen I proteins in the alveolar bone tissue collected in 4 weeks. Notably, MT01 treatment induced much more OPG expressing and Osterix expressing cells in the tissue. The results demonstrated that MT01 mediated prevention of alveolar bone absorption in the rat with periodontitis was associated with OPG and Osterix production.

## 3. Experimental Section

### 3.1. Materials

The ODNs were synthesized by TaKaRa (Dalian, China). The sequences of ODNs were as follows: FC003 (5′-TCT CTC TCT CTC TCT CTC TCT CTC-3′), SAT05f (5′-CCT CCT CCT CCT CCT CCT CCT CCT-3′) [29], SAT05d (5′-CTC TCT CTC TCT CTC TCT CTC TCT-3′) [29], MS19 (5′-AAA GAA AGA AAG AAA GAA AGA AAG-3′) [32], BW001 (5′-TCG TCG GGT GCG ACG TCG CAG GGG GG-3′) [31], FC001 (5′-TCG GGG ACG ATC GTC GGG GGG-3′) [33], FC002 (5′-TCG TCG ACG TCG TTC GTT CTC-3′) [34], BW006 (5′-TCG ACG TTC GTC GTT CGT CGT TC-3′) [33], YW002 (5′-TCG CGA ACG TTC GCC GCG TTC GAA CGC GG-3′) [32], YW001 (5′-TCG CGA CGT TCG CCC GAC GTT CGG TA-3′) [34], FC004 (5′-TCG CGA ACG TTC GCC CGA TCG TCG GTA-3′) [31] and MT01 (5′-ACC CCC TCT ACC CCC TCT ACC CCC TCT-3′) [29]. All ODNs were diluted using PBS buffer, and no endotoxins were detected (Limulus amebocyte lysate assay, Associates of Cape Cod, Inc.). All the reagents used in the research were pyrogen-free.

### 3.2. Isolation, Culture and Identification of BMSCs

One-month rats were sacrificed by cervical dislocation, and sterilized with alcohol of 0.75 volume fraction for 10 min. The femur and tibia were removed, and the osteoepiphysis was sheared off. The bone marrow was collected using a syringe with 5 mL of serum-free DMEM. The suspension was transferred into a centrifuge tube, blown and centrifuged at 1000 r/min for 5 min. After discarding the supernatant and adipose layer, 5 mL DMEM containing 10% fetal calf serum was added to re-suspend the cells. The cells at density of 1 × 10^9^ L^−1^ were inoculated in a 25 cm^2^ culture flask and cultured at 37 °C in the 5% CO_2_ incubator at saturated humidity. After 48 h, half of the medium was removed and replaced with fresh medium. The cells were further cultured for 3 days, and then the medium was completely replaced to remove the non-adherent cells. Afterwards, the medium was completely replaced every 3 days. The cells were observed under an inverted microscope daily. When 80–90% confluence was achieved, the cells were digested with 0.25% trypsin containing 0.02% EDTA and passaged at a ratio of 1:2. The cells at the third passage were harvested to prepare the single cell suspension and then identified by staining with phyco-erithrin conjugated antibodies (Sangon Biotech., Shanghai, China) against CD34, CD44, CD45, CD90 and CD116, respectively, and analyzed on a FACS caliber (Becton Dickinson, San Jose, CA, USA) using Cell Quest software with 10,000 events collected for each sample. The identified CD45, CD90 and CD116 positive cells were used as BMSCs [40].

### 3.3. Proliferation Assay

Proliferation of BMSCs was determined using 3-(4,5-dimethylthiazol-2-yl)-2,5-diphenyltetrazolium bromide (MTT) assay as previously described [41]. Briefly, 5 × 10^3^ cells/well in 200 μL DMEM containing 10% fetal calf serum were inoculated into 96-well plates and cultured with medium containing various ODNs at a final concentration of 1 μg/mL. The cells were incubated at 37 °C for 72 h, and 10 μL of MTT solution (5 mg/mL) was added, followed by incubating for another 4 h. Finally, the supernatant was removed, and cells were lysed with 150 μL DMSO. The absorbance at 570 nm of each well was measured using a Bio RAD 550 automatic plate reader.

### 3.4. ALP Analysis in BMSCs

BMSCs at 2 × 10^4^ cells/well at the third passage were cultured in 1000 μL DMEM containing 10% fetal calf serum in a 24-well plate for 24 h, and then the medium was completely replaced with fresh medium with a 1% osteogenesis inducing solution containing 10 mmol/L β-glycerin phosphoric acid, 0.05 mmol/L vitamin C and 100 mmol/L dexamethasone in PBS. After the addition of ODNs at a final concentration of 1 μg/mL, the cells were cultured for 72 h, and washed with PBS twice. The washed cells were lysed at 0 °C for 30 min. Total protein concentration and ALP activity in the cell lysate were measured using a micro-BCA assay kit (Jiancheng Biological Reagent Co., Nanjing, China) and an alkaline phosphatase kit (Jiancheng Biological Reagent Co., Nanjing, China) according to the manufactures’ instructions, respectively. The average ALP activity of triplicate measurements was normalized with the reference of total protein concentration.

### 3.5. Quantitative Real-Time PCR

Total RNA was isolated from BMSCs using the Trizol reagent (Sigma, St. Louis, MO, USA) according to the manufacturer’s instructions. The purity of total RNA was determined by the ratio at 260 nm and 280 nm absorbance. Using RT-PCR Array First Strand Kit (SA Bioscience Co., Carlsbad, CA, USA), 1 μg of total RNA was reverse-transcribed into total cDNAs. The cDNAs were subjected to PCR amplification by using specific primers as follows: RANKL (F: 5′-CTG ATG AAA GGA GGG AGC AC-3′; R: 5′-GAA GGG TTG GAC ACC TGA ATG C-3′), OPG (F: 5′-TCC TGG CAC CTA CCT AAA ACA GCA-3′; R: 5′-ACA CTG GGC TGC AAT ACA CA-3′), Osterix (F: 5′-CCT CTC GAC CCG ACT GCA GAT C-3′; R: 5′-AGC TGC AAG CTC TCT GTA ACC ATG AC-3′), Runx2 (F: 5′-TGC TTC ATT CGC CTC ACA AA-3′; R: 5′-TTG CAG TCT TCC TGG AGA AAG TT-3′ ) and collagen I (F: 5′-GAG ATG ATG GGG AAG CTG-3′; R: 5′-ACC ATC CAA ACC ACT GAA G-3′). In each sample, the specific primers of β-actin (F: 5′-TCA GGT CAT CAC TAT CGC CAA T-3′; R: 5′-AAA GAA AGG GTG TAA AAC GCA-3′) were used as an internal reference and simultaneously amplified. Efficiencies (E) of amplicons in the PCRs were determined by dilution curves, and used to calculate the relative quantification values for each target cDNA. The PCRs were performed using an ABI PRISM 7000 detection system under the conditions as follows: 50 °C for 2 min, 95 °C for 10 min, 45 cycles of 95 °C for 15 s, and 60 °C for 1 min. The cycle threshold (Ct) was established in the linear part of the reaction curve at which the amount of amplified target cDNA was calculated. The relative expression levels of a target gene were normalized with the value of β-actin gene, and the 2^−ΔΔCt^ method [42] was used for calculating the relative expression levels. The values were the average of triplicate measurements.

### 3.6. Rat Experiment

Six-week-old specific pathogen-free (SPF) male Wistar rats (180–210 g body weight) were purchased from Animal Center of Norman Bethune Medical College, Jilin University (Changchun) and maintained in laminar flow rooms and used for experiments in accordance with the National Institute of Health Guide for the Care and Use of Laboratory Animals, and with the approval of the Scientific Investigation Board of Science and Technology of Jilin Province. To establish a periodontitis model, rats were intramuscularly anesthetized with 0.8 mL/kg body weight of Su-Mian-Xin (Veterinary Institute of Military Supplies University, Changchun, China), composed of dihydroetorphine hydrochloride, dimethylaniline thiazole, EDTA and haloperidol. Sterile, 3–0 black braided nylon thread (surgilon; USS/DG, Norwalk, CT, USA) was placed around the cervical margins of the bilateral second maxillary molars and knotted mesially. The ligature was knotted on the buccal side of the tooth, resulting in a subgingival position palatal and a supragingival position buccally, as previously described [43].

On days 0, 3, 5, and 7 after the ligature placement, the rats were injected with MT01 (20 μg/kg) or PBS in the gingiva of the left and right second maxillary molars, respectively. At 2 and 4 weeks after the ligature placement, 6 rats in each group were euthanized. One side of the maxillary of the rats was removed and stored at −80 °C for routine histological examination, and the other side was stored at −20 °C for measuring its alveolar bone loss.

### 3.7. Microscopic Examination of Alveolar Bone Loss

To measure the alveolar bone loss, frozen maxillaries were thawed, de-fleshed by 2 N NaOH for 10 min, then washed and air dried. Each maxillary was fixed with white wax in an orientation to make buccal and lingual cusps, superimposed for taking photos using a Stereoscope (Stemi SV 11; Zeiss, Oberkochen, Germany) equipped with a videocamera (Axio-Cam HRc; Zeiss) and a TV monitor, which displayed the distance digitally. The photos were analyzed by SPOT RT software v3.5 [38,39]. To reduce the possible errors caused by the projection angle, a graduated periodontal probe was photographed alongside the maxillaries. The distances from the amelocemental junction to the alveolar crest were recorded six times per tooth. The average distance (expressed in mm) for each tooth was used as a measurement of bone loss. The sensitivity of the measurement was *ca.* 10. These measurements were repeated three times at one week intervals. The average coefficient of variation for the measurements was 1.53%.

### 3.8. Immunohistochemistry of RANKL, OPG, Osterix, Runx2 and Collagen I

Thin sections of periodontal tissue (5 μm) were obtained by using a microtome and transferred to a gelatin coated slide. The tissue section was first deparaffinized and then rehydrated. The gingival and periodontal tissue slices, after washing with 0.3% Triton X-100 in phosphate buffer, and quenching of endogenous peroxidase (3% hydrogen peroxide), were incubated with primary antibody (RANKL 1:250, or OPG 1:250, or Runx2 1:250, or Osterix 1:250, or collagen I 1:250) overnight at 4 °C. After washing with phosphate buffer, the slices were incubated with secondary antibody for 1 h, the immuno-reactivity to RANKL, OPG, Osterix, Runx2 and collagen I was visualized using a colorimetric-based detection kit following the manufacturer’s protocol (Dako LSAB + Kit, peroxidase, DAKO, Carprinteria, CA, USA). The positive cells expressing RANKL, OPG, Osterix, RunX2 or collagen I were numerated under a microscope (Olympus Optical Co. Ltd, Tokyo, Japan) using 200× objective in a double-blind manner. The results were presented as mean numbers of cell/field, which were obtained by counting nine randomly selected microscopic fields (0.006 mm^2^/each) with the gingival tissue between the first and second molars.

### 3.9. Statistical Analysis

All data were presented as the mean ± SD. The significance of the differences was determined using the two-tailed Student’s *t* test and one-way ANOVA. For each figure, representative results from 2–3 independent experiments were exhibited.

## 4. Conclusions

In this study, MT01, a synthetic ODN, designed with the reference of the sequence of human mitochondrial DNA, has been shown to be able to induce differentiation of BMSCs to osteoblasts *in vitro* and prevent alveolar bone absorption in rats with periodontitis. The data suggest that MT01 could be developed as a candidate agent for the treatment of periodontitis by reducing alveolar bone absorption.
